# Stay-at-home, *Safe at Home*? A survey of parental home safety practices before and during the COVID-19 pandemic

**DOI:** 10.1186/s40621-022-00396-4

**Published:** 2022-12-21

**Authors:** Kristin J. Roberts, Rebecca J. McAdams, Lara B. McKenzie

**Affiliations:** 1 Center for Injury Research and Policy, The Abigail Wexner Research Institute at Nationwide Children’s Hospital, 700 Children’s Drive, Research Building III, Columbus, OH 43205 USA; 2 Department of Pediatrics, The Ohio State University, College of Medicine, 370 W. 9th Ave., Columbus, OH 43210 USA; 3 Division of Epidemiology, The Ohio State University, College of Public Health, 250 Cunz Hall, 1841 Neil Ave., Columbus, OH 43210 USA

**Keywords:** COVID-19, Stay-at-home orders, Unintentional pediatric injury, Safety devices, Injury prevention practices

## Abstract

**Background:**

To slow the spread of COVID-19, many nonessential businesses, daycares, and schools closed, and areas imposed “stay-at-home” orders. Closures led to young children spending more time at home, traditionally, the place where more than one-half of unintentional pediatric injuries occur. The objective of the current study was to describe parental safety perceptions and confidence, safety device purchase and installation, and injury prevention practices and behaviors, in homes with children 6 years of age and younger, before and during the COVID-19 pandemic.

**Methods:**

A cross-sectional survey with a convenience sample of US participants, 18 years or older, was conducted from November 2020 to February 2021. Parents of children (≤ 6 years) were recruited via social media ads and posts on Facebook and Twitter and invited to complete an anonymous, online survey about their home safety practices before and during the COVID-19 stay-at-home order. Upon completion, parents could participate in a prize drawing to receive one of five $100 gift cards.

**Results:**

A total of 499 participants completed the survey. Most (47.9%) were 45–54 years of age and reported the amount of time at home increased for them (93.9%) and their children (90.6%) during the stay-at-home period. Thirty-seven percent (36.9%) of parents considered their homes safe but recognized room for improvement and felt confident in their ability to make their homes safe for their children (72.8%). From the time before until the COVID-19 stay-at-home orders were in place, parents increased their home injury prevention practices (42.3%). Parents that had identified unsafe areas in the home before the stay-at-home order were significantly more likely to increase their safety behaviors, take childproofing actions, and purchase or install safety devices during the stay-at-home order (*p* < 0.0001). Parents with younger children (5 years) were significantly more likely than parents with older children to take childproofing actions (*p* < .0001) including purchasing and installing safety devices (*p* < 0.0001).

**Conclusions:**

Spending more time at home during the COVID-19 pandemic may have helped the sampled parents, especially those with younger children, identify unsafe areas in their homes and encourage them to modify their behaviors, and purchase and install safety devices to help make their homes safer for their children.

**Supplementary Information:**

The online version contains supplementary material available at 10.1186/s40621-022-00396-4.

## Background

COVID-19 (coronavirus disease) is an infectious disease that led to the first major pandemic of our generation (World Health Organization 2021a). In 2020, the virus had spread worldwide and impacted nearly every aspect of daily life including healthcare, education, transportation, and religious and cultural activities. As of September 2021, more than 41 million cases have been reported in the USA resulting in over 660,000 deaths (Centers for Disease Control and Prevention 2021a; World Health Organization 2021b). To slow the spread of the virus, many areas closed nonessential businesses and imposed “lockdown” or “stay-at-home” (SAH) orders for varying periods of time (Czeisler et al. 2020; Centers for Disease Control and Prevention 2021b). When schools and daycare centers were closed, children and families were displaced to spend more time in the home, where unintentional child injuries can occur. The SAH orders were intended to limit the spread of the COVID-19; however, the impact these orders would have on child injury prevention practices and child injury in the home was unknown.

In the USA, unintentional injury, including burns, poisonings, and falls, is the leading cause of death for children (National Vital Statistics System 2018) resulting in over 12,000 deaths annually (Borse et al. 2008). More than 50% of these injuries occur in and around the home, where young children spend most of their time (Bergen et al. 2007). Interestingly, many of these injuries can be prevented through the correct use and installation of safety equipment and caregiver adherence to existing safety recommendations (Kendrick et al. 2005; Stewart et al. 2016). Although known countermeasures exist for pediatric injury prevention, historically, the use of devices and safety behaviors has been low and varied by countermeasures (Safe Kids Worldwide 2015). Parents play a critical role in the prevention of home injuries, yet barriers may include the ability to identify home hazards (Gaines and Schwebel 2009), as well as time and money needed to obtain and install safety devices in the home (Pomerantz et al. 2016; Gielen et al. 1995; Roberts et al. 2019). Successful prevention relies on parents’ ability to change their behavior and modify their environment to make their homes safer for their children (Pomerantz et al. 2016; Russell et al. 2013; Kendrick et al. 2013). While the stay-at-home orders may have slowed the spread of the disease, many parents became faced with new challenges and stressors such as unemployment, working from home, providing education/homeschooling, and solely caring for their child(ren) as schools and daycares closed (Spinelli et al. 2020). Although pediatric emergency department visits declined during the stay-at-home period (Sethuraman et al. 2021; Pines et al. 2021), time spent at home with children may have contributed to more home injuries (Gielen et al. 2020). However, very little is known about how the stay-at-home orders impacted parental home safety prevention awareness, decisions, and practices.

Therefore, the goal of this study was to describe parental safety perception and confidence regarding home safety, safety device acquisition and installation, and injury prevention practices and behaviors, in homes with children 6 years and younger, before and during the COVID-19 pandemic.

## Methods

### Study design

A cross-sectional survey with a convenience sample of US parents and caregivers ($\ge$18 years) of young children (≤ 6 years of age) were invited to complete an online, anonymous survey (using SurveyMonkey^®^), entitled *Safe at Home*, about their home safety practices before and during the beginning of the COVID-19 pandemic. The survey was fielded from November 2020 through February 2021.

### Recruitment

Participants were recruited through social media (i.e. Facebook and Twitter) posts and electronic flyers sent via email. Posts and flyers contained a brief description of the study and included a direct link for interested participants to determine eligibility and to complete informed consent prior to completing the survey. The social media posts and flyer contained (1) a black and white image of a woman using a tablet, (2) a short title inviting the parents to “help researchers learn more about home safety before and during COVID-19,” and (3) a series of short titles and descriptions telling the potential participant about the purpose of the study, eligibility requirements, a description of what is expected to participate in the study and a link to the survey. Additionally, the posts and flyer contained the contact information of the study’s principal investigator and were branded with Nationwide Children’s Hospital’s logo. The electronic flyer was shared with friends and colleagues via email inviting them to share the invitation to participate among their networks while the social media post was shared four times over the course of the study on the Nationwide Children’s Hospital’s Twitter and Facebook pages.

### Online survey

Interested participants were able to click a link to the online survey after completing eligibility questions and online consent. To be eligible, participants were required to be (1) ≥ 18 years of age, (2) a parent, caregiver, or legal guardian of at least one child ≤ 6 years of age who lives with them most of the time, and (3) living in the US or US territory most of the time. The Safe at Home Survey (e.g., Additional file 1) contained 36 questions and took an average of 9 min to complete.

Upon completion of the survey, participants had the opportunity to enter a drawing for a $100 gift card. Prize drawings were awarded for every 100 completed surveys (*n* = 5). This study was approved by the Institutional Review Board at Nationwide Children’s Hospital, Columbus, Ohio.

## Measures

The goal of this study was to describe (1) parental perception of home safety and parental safety confidence, and (2) injury prevention practices and behaviors including safety device acquisition and installation in and around the home before and during the COVID-19 SAH orders. The survey consisted of a combination of yes–no, multiple choice, and Likert-scale response items.

### Demographic characteristics

Basic demographic information including participants’ gender, age, education and employment status, and number and age of their children, were collected. The age of the respondent’s children was categorized into three groups: (1) younger children only (i.e. all children for each participant were < 5 years of age); (2) older children only (i.e. all children for each participant were ≥ 5 years of age); and (3) younger and older children only (i.e. each participant had children in both the < 5 years and ≥ 5 years age-groups. Participants were asked if the status of the following items had changed because of the pandemic including the time they and their children spent at home: childcare, homeschooling, ability to work from home, moved households, and the burden of the pandemic on household finances.

### Parental perception of home safety

Participants were asked how safe they considered their homes to be at preventing injury for their child(ren) before and during the SAH order using a Likert scale (1 through 5); (1) not safe, a lot of room for improvement, (3) safe, but room for improvement, or (5) extremely safe. A variable to categorize a change in parental perception of home safety variable was also created to show how safe participants considered their home to be at preventing injury for their child(ren) from injury before and during the SAH order; (1) no change, (2) considered home *more* safe during the SAH order, or (3) considered home *less* safe during the SAH order.

Participants were also asked if they had identified areas in their home that may be unsafe for their child(ren) before and during the COVID-19 pandemic (yes–no). A variable was created to illustrate if participants noticed areas in their home that may be unsafe before to children during the SAH order. This change in the unsafe areas noticed variable had three categories: (1) no change in areas noticed, (2) noticed more unsafe areas in the home during the stay-at-home order, or (3) noticed fewer unsafe areas in the home during the stay-at-home order.

### Parental safety confidence

Confidence in home safety was assessed. Participants selected whether they were (a) extremely confident, (b) very confident, (c) moderately confident, (d) slightly confident, or (d) not at all confident in their ability to accomplish six tasks: (1) follow home safety recommendations, (2) take actions to make their home as safe as possible, (3) know the home injury hazards, (4) purchase the correct home safety products and devices needed, (5) correctly install the safety products and devices in their home, and (6) know what to do to make their home as safe as possible, and know that they are taking safety actions that will keep their child(ren) from being injured in and around their home. Each answer was dichotomized and a new Parental Safety Confidence variable created: (1) confident (which included extremely confident and very confident); (2) not confident, (which included moderately confident, slightly confident, and not at all confident).

### Injury prevention practices and behaviors

Participants were asked if their safety behaviors (e.g., locking up medicines, turning pot handles to the back of the stove when cooking) had changed during the SAH order compared to before the SAH order (no change in safety behaviors; yes, safety behaviors increased; yes, safety behaviors decreased). Participants were asked if they had taken any additional steps to childproof their home (yes, no) and if they had purchased or installed any safety products (yes, no) since the beginning of the SAH order.

### Safety device acquisition and installation

Participants were asked to indicate the safety products (i.e. smoke alarms, carbon monoxide alarms, cabinet locks or latches, stair gates, room gates and barriers, TV wall mount or anti-tip straps, door knob covers, window stops or locks, and other safety products) they had in their home before SAH order and if they had installed any safety products in their home after the SAH order was in place. The change in safety device installation from before the SAH to during the SAH order variable was created: (1) installed device: no device or not applicable before the SAH and installed a needed device or another device since the beginning of the SAH; (2) did not install a needed device: no device before the SAH and did not install a device since the beginning of the SAH; and (3) device install not needed: had a device so an install was not needed.

### Statistical analysis

The data were analyzed by using SAS version 9.4 (SAS Institute, Inc., Cary, NC). Bivariate comparisons were performed using chi-square tests. A Wilcoxon signed-rank test was used to test the difference in median score indicating how safe participants considered their home at preventing child injury before versus during the SAH order. Relative risks with their associated 95% CIs were generated to assess the strength of association between the child age-group and taking additional steps to childproof the home as well as purchasing or installing any safety device since the beginning of the SAH order.

We performed multivariate logistic regression; for each model, the independent variable was noticing areas that were unsafe in the home before the SAH order. After a backward selection process with all potential confounders in the models, all models were adjusted for child age-group, work-from-home status, and essential worker status to determine the odds of (1) safety behaviors increasing, (2) additional steps to childproof the home being taken, and (3) purchasing and installing safety devices once SAH orders were in place. For all models, the odds ratio and corresponding 95% CI were calculated. Statistical significance was set at alpha = 0.05.

## Results

During the study period, the recruitment social post was shared four times on Nationwide Children’s Hospital’s Facebook page, resulting in a reach of over 53,000 yielding a total of 442 link clicks. The social post was also shared eight times by Nationwide Children’s Hospital’s Twitter account yielding a total of 23,974 impressions or views and 54 link clicks.

A total of 596 individuals started the *Safe at Home* survey. Of those, 7 did not consent to participate, 54 individuals did not provide an age over 18 years, and 13 did not have a child ≤ 6 years of age who lives with them most of the time, yielding a sample of 522 participants who consented to participate and completed the survey. Additionally, three surveys were excluded because the participant selected only children ages over 7 years. Two surveys were excluded because the participant did not provide exact child age information. Finally, twelve surveys were excluded because they were missing more than 90% of the survey responses, and six were excluded because the participant completed the survey in less than 1 min, a strong indication that the questions were not read or answered with intention. A total of 499 completed surveys were included in the final analyses.

### Demographic characteristics

The majority of the 499 participants were 45–54 (47.9%) and 35–44 (45.1%) years of age with only younger (< 5 years) children (54.5%). There were 849 children among the participants. Of all the participants, 21.6% had a child 1 year of age, 26.5% had a child 2 years of age, and 21.2% had a child 3 years of age. Most participants were employed full time (54.2%) before and during (43.9%) the stay-at-home order and about one-half (56.1%) were working from home at the time of the survey. Almost all (93.9%) of participants reported their time at home had increased and 90.6% reported the amount of time their child(ren) spent at home increased during the SAH. Of those who had at least one child over 5 years of age (i.e. older children only or mix older/younger), 72.0% homeschooled at least one child during the stay-at-home order. Since the start of the SAH, 17.0% of our participants reported moving homes. When asked to think about their household’s total income, 34.1% of the participants reported the COVID-19 pandemic being a moderate or substantial financial burden to their families (Table 1).

**Table 1:** Characteristics of *Safe at Home* survey participants

	*n*^a^	%
Total number of participants	499	100.0
*Parent age (years)*		
18–24	0	0.0
25–34	14	2.8
35–44	225	45.1
45–54	239	47.9
55 +	21	4.2
Total number of children	849	100.0
*Child age (years)*		
< 1	100	20.0
1	108	21.6
2	132	26.5
3	106	21.2
4	110	22.0
5	89	17.8
6	107	21.4
7	97	19.4
*Child age*		
Younger only (< 5 years)	272	54.5
Mix younger and older	149	29.9
Older only (≥ 5 years)	78	15.6
*Parent time in home*		
Increased	458	93.9
Decreased	12	2.5
Did not change	18	3.7
*Child(ren) time in home*		
Increased	441	90.6
Decreased	16	3.3
Did not change	30	6.2
*Working from home*		
Yes	236	56.1
Somewhat	75	17.8
No	93	22.1
Other^b^	17	4.0
*Pandemic financial burden*		
No/not much	282	65.9
Moderate/substantial	146	34.1
*Homeschooling*		
No	233	54.3
Yes	193	45.0
Prior to SAH order	3	0.7
*Moved homes*		
No	390	83.0
Yes	80	17.0

^a^Some categories do not total 499 because missing values were not included

^b^Other includes stay-at-home parent and maternity leave

### Parental perception of home safety

When asked to think about their home’s safety before the SAH order, parents most frequently (36.9%) considered their homes to be safe but agreed there was room for improvement. There was no difference (*p* = 0.0969) in the median score of how safe participants considered their homes to be at preventing injury for their child(ren) before (median, 4) and during the SAH order (median, 4). Over 75% (76.5%) of participants had no change in how safe they considered their homes to be before and during the SAH order. A total of 13.6% of participants viewed their homes as safer during the SAH order compared to before, whereas 9.9% of participants believed theirs home to be less safe during the SAH order compared to before (Table 2).

**Table 2:** Parental perception of home safety, overall and by child age-group

	*n*^a^	%	Younger only		Older only		Mix older and younger		*p* value
			*n*	%	*n*	%	*n*	%	
Perceived perception of home safety									
*Considering home safe*									0.0430
No change	365	76.5	188	72.0	65	87.8	112	78.9	
Considered home more safe during SAH order	65	13.6	40	15.3	5	6.8	20	14.1	
Considered home less safe during SAH order	47	9.9	33	12.6	4	5.4	10	7.0	
*Noticing unsafe areas of the home*									0.3048
No change	309	69.3	160	65.8	53	74.7	96	72.3	
No change: safe	166	n/a	n/a	n/a	n/a	n/a	n/a	n/a	
No change: unsafe	143	n/a	n/a	n/a	n/a	n/a	n/a	n/a	
Noticed more unsafe home areas during SAH order	34	7.6	22	9.1	2	2.8	10	7.6	
Noticed less unsafe home areas during SAH order	103	23.1	61	25.1	16	22.5	26	19.7	

^a^Some categories do not total 499 because missing values were not included

There was a significant association between the change in home safety perception and the child age-group (*p* = 0.0430). A total of 72.0% of participants with only young children while 87.8% of participants with only older children had no change in their parental perception of home safety from before to during the SAH order. Fifteen percent (15.3%) of participants who only had younger children and 6.8% of participants who only had older children considered their homes to be safer during the SAH order compared to before it (Table 2).

Before the SAH order, 53.2% of participants noticed areas in their homes that may be unsafe for their children. Since the beginning of the stay-at-home order, most participants did not notice additional areas in their homes that may be unsafe for their children (59.3%). The majority of participants (69.3%) did not experience a change in noticing unsafe areas in their homes since before to during the SAH order. Over twenty percent (23.1%) of participants reported noticing fewer unsafe areas in their homes since the beginning of the SAH order compared to before the SAH order, while 7.6% of participants reported noticing more unsafe areas in their homes since the beginning of the SAH order compared to before it. There was a significant relationship between the areas noticed to be unsafe in the home before and since the beginning of the SAH order (*p* < 0.0001). There was no difference by child age-group in areas participants noticed to be unsafe in the home from before to during the SAH order (*p* = 0.3048) (Table 2).

### Parental safety confidence

Participants reported high levels of confidence regarding knowing common home injury hazards (65.2%), purchasing (71.5%) and installing (64.6%) safety products, knowing what to do to keep their homes safe (63.9%), and taking actions to prevent home injury (72.4%). Participants were more confident in their abilities to take actions to make their home as safe as possible (72.8%) while participants were least confident (49.9%) in their ability to do everything that is recommended by home safety experts (Table 3). Taking actions to make their home as safe as possible differed by child age-group (*p* = 0.0157). Participants who only had younger children yielded the smallest percent of participants who were very confident or extremely confident that they can make their home as safe as possible (67.6%). Participants who only had older children had the highest percent of confidence that they can make their home as safe as possible (83.6%).

**Table 3:** Parental safety confidence for *Safe at Home* survey participants

Parental safety confidence questions	Not confident^a^		Confident^b^	
	*n*^c^	%	*n*^c^	%
I am__that I do everything that is recommended by home safety experts	220	50.1	219	49.9
I am__that I can take actions to make my home as safe as possible	119	27.2	319	72.8
I am__that I know the most common home injury hazards	152	34.8	285	65.2
I am__that I can purchase the exact home safety products and devices I need	125	28.5	314	71.5
I am__that I can correctly install the safety products and devices in my home	155	35.4	283	64.6
I am__that I know what to do to make my home as safe as possible	158	36.1	280	63.9
I am__that taking safety actions will keep my child from being injured in and around my home	121	27.6	318	72.4

^a^Not confident includes moderately confident, slightly confident, and not at all confident

^b^Confident includes extremely confident and very confident

^c^Some categories do not total 499 because missing values were not included

### Injury prevention practices

While most participants (53.6%) did not change their injury prevention behaviors, over forty percent (42.5%) reported increasing their injury prevention behaviors during the SAH period. The majority of participants with younger children only (52.8%) reported that their injury prevention behaviors increased, while the majority of participants with older children only (71.6%) and participants with both younger and older children (66.2%) reported that their injury prevention behaviors did not change during the SAH order (*p* < 0.0001) (Table 4). Participants who noticed unsafe areas in the home before the SAH order were 2.6 times (OR = 2.60; 95% CI 1.66, 2.06) more likely to increase their safety behaviors during the SAH order than those who did not notice unsafe areas before the SAH order, after adjusting for child age-group, work-from-home status, and essential worker status (Table 5, Model 1).

**Table 4:** Injury prevention practices, overall and by child age-group

	*n*^a^	%	Younger only		Older only		Mix older and younger		*p* value
			*n*	%	*n*	%	*n*	%	
Injury prevention practices									
*Safety behaviors changed during SAH order*									< 0.0001
No change in behaviors	251	53.6	104	41.3	53	71.6	94	66.2	
Yes, safety behaviors increased	199	42.5	133	52.8	20	27.0	46	32.4	
Yes, safety behaviors decreased	18	3.9	15	6.0	1	1.4	2	1.4	
*Additional steps taken to childproof home*									< 0.0001
No	270	57.7	116	45.7	65	87.8	89	63.6	
Yes	198	42.3	138	54.3	9	12.2	51	36.4	
*Purchased or installed any safety device for home*									< 0.0001
No	285	61.0	128	50.8	66	89.2	91	64.5	
Yes	182	39.0	124	49.2	8	10.8	50	35.5	

^a^Some categories do not total 499 because missing values were not included

**Table 5:** Adjusted odds ratios with noticing unsafe areas in the home as exposure

Model^a^	Odds ratio	95% CI	Wald statistic	*p* value
Model 1^b^	2.60	1.66, 4.06	17.49	< 0.0001
Model 2^c^	4.14	2.61, 6.58	36.19	< 0.0001
Model 3^d^	3.90	2.43, 6.26	31.74	< 0.0001

Statistical sign at alpha = 0.05

^a^Every model is adjusted for child age-group, work-from-home status, and essential worker status

^b^Model 1 had the outcome of during the SAH order safety behaviors increased (dependent variable)

^c^Model 2 had the outcome of taking additional steps to childproof home since beginning of SAH order (dependent variable)

^d^Model 3 the outcome was whether a parent purchased or installed any safety device since the beginning of SAH order

### Additional steps to childproof the home

Since stay-at-home orders were implemented, 42.3% of participants took steps to childproof their homes and 39.0% of participants purchased or installed at least one safety device (Table 4). There was a significant association between the child age-group and those who took additional steps to childproof their homes since the beginning of the SAH order (*p* < 0.0001). More than half (54.3%) of participants with only younger children took additional steps to childproof their home since the beginning of the SAH order, followed by participants with both younger and older children (36.4%), and participants with only older children (12.2%). Participants with only younger children had almost twice the likelihood of taking additional steps to childproof their home during the SAH order than participants with both younger and older, and only older children. RR = 1.95 (95% CI 1.52, 2.47). Participants with at least some younger children (younger children only and those with both younger and older children) had almost four times the likelihood (RR = 3.95; 95% CI 2.12, 7.34) of taking additional steps to childproof their home during the SAH order than participants with only older children. Participants who noticed unsafe areas in the home before the SAH order were 4.14 times more likely (95% CI 2.61, 6.58) to take additional steps to childproof the home during the SAH order than those who did not notice unsafe areas in the home before the SAH order, adjusted for child age-group, work-from-home status, and essential worker status (Table 5, Model 2). Over fifty percent (51.9%) of the participants who moved during the SAH order took additional steps to childproof their homes (*p* < 0.0584).

### Purchased or installed safety devices

About one-half (49.2%) of participants with only younger children purchased or installed safety devices since the beginning of the SAH order, followed by participants with both younger and older children (35.5%), and participants with only older children (10.8%). Participants who had only younger children were significantly more likely (*p* < 0.0001) to purchase or install safety devices in their homes since the beginning of the SAH. In fact, participants with only younger children had almost twice the likelihood of purchasing or installing safety devices since the beginning of the SAH order compared to participants with both younger and older children, and those with only older children RR = 1.82 (95% CI 1.42, 2.35). Participants with some younger children (younger only children and also those with both younger and older children) were four times more likely (RR = 4.10; 95% CI 2.11, 7.95) to purchase or install safety devices since the beginning of the SAH order compared to those participants with only older children.

Over fifty percent (54.4%) of participants who moved during the SAH order purchased or installed at least one safety device. Participants that reported moving during the pandemic were more likely to purchase or install safety devices than those that did not move homes (*p* < 0.002).

### Safety device acquisition and installation

Participants were asked to report the safety devices in their homes before the SAH order. Participants most frequently reported having smoke alarms (96.4%) and carbon monoxide alarms (84.8%) in their homes before the SAH order. When asked to report the products they had installed in their home since the beginning of the SAH, participants most often reported installing gates or barriers (33.5%), cabinet locks and latches (31.4%), and TV mounts (27.8%) (Table 6). Of those participants that purchased and installed a device during the SAH that they didn’t have before the SAH, the majority were participants with younger children only. For example, 66.4% of participants who reported installing a smoke alarm and 66.7% of participants who reported installing a carbon monoxide alarm since the beginning of the SAH order had only younger children (*p* = 0.0005 and *p* = 0.0025, respectively). For each safety device, participants with only younger children were significantly more likely to purchase and install a safety device during the SAH compared to participants with older children only or a mix of younger and older children living in the home (Table 6). Participants who noticed unsafe areas in the home before the SAH order were 3.90 times more likely (95% CI 2.43, 6.25) to purchase or install a safety device during the SAH order than those who did not notice unsafe areas in the home before the SAH order, adjusted for child age-group, work-from-home status, and essential worker status (Table 5, Model 3).

**Table 6:** Safety device installation before and since the beginning of the COVID-19 stay-at-home order, overall and by child age-group

	Before the stay-at-home order		Since the beginning of the stay-at-home order								
	Devices in the home		Installed device		Younger children only		Older children only		Mix of younger and older		*p* value
	*n*^a^	%	*n*^a^	%	*n*	%	*n*	%	*n*	%	
Smoke alarms	478	96.4	113	24.5	75	66.4	8	7.1	30	26.6	0.0005
CO alarms	420	84.8	117	25.6	78	66.7	8	6.8	31	26.5	0.0025
Cabinet locks/latches	282	57.4	136	31.4	99	72.8	4	2.9	33	24.3	< 0.0001
Gates/barriers	305	61.6	139	33.5	103	74.1	3	2.2	33	23.7	< 0.0001
TV mount	300	61.0	118	27.8	87	73.7	3	2.5	28	23.7	< 0.0001
Doorknob covers	143	29.0	84	21.3	65	77.4	1	1.2	18	21.4	< 0.0001
Window stops	310	62.8	86	20.0	56	65.1	4	4.7	26	30.2	0.002
Other	261	58.9	108	31.0	68	63	8	7.4	32	29.6	0.0098

^a^Some categories do not total 499 because missing values were not included

## Discussion

This study is one of the first to examine parental safety perception and child injury prevention practices in and around the home for parents with young children (≤ 6 years of age) during the COVID-19 pandemic. In early 2020, COVID-19 emerged in the US as the first global pandemic of our time causing businesses to close and encouraging people to stay-at-home. While the stay-at-home orders were put in place to slow the spread and reduce the potential for COVID-19 infection, under the orders parents and children began spending more time at home, where unintentional child injuries are more likely to occur. Although most home-related injuries are preventable, prevention requires parental awareness of injury hazards, parental confidence, and the ability to make their home a safer space for their children. Sampled parents reported high levels of confidence in their ability to make their home safer for their children and were aware that unsafe areas may exist in their home. During the SAH order, sampled parents, especially those with younger children, took additional steps including increasing their safety behaviors as well as purchasing and installing safety devices to make their homes safer for their young children.

We found that before the SAH order, most parents considered their homes to be safe, but recognized there was room for improvement. The majority of parents in this study were confident in their ability to identify home injury hazards and take the steps needed to make their homes safer. However, the age of the children was associated with parental safety confidence as parents with only younger children were less confident in their ability to make their home safe. For parents with younger children, preventing child injuries in and around the home may feel like a daunting task as there are many areas in the home which can lead to child injury. This may feel overwhelming, especially to new parents who may not have previously thought about child injury prevention and may never have needed to childproof a home.

Although parents with only younger children were less confident in their ability to make their homes safer, they were nearly four times more likely to take action to childproof their homes when compared to parents with only children ≥ 5 years of age. In addition to taking action and childproofing their homes, the sampled parents with only younger children were significantly more likely to purchase and install safety devices. It is recommended that parents take safety measures to prevent injury before the child is developmentally able to explore the injury hazard. To prevent injuries in the home, childproofing should occur before the child begins crawling or walking (American Academy of Pediatrics 2019a) and may continue through the preschool years as injury risk shifts to being child-initiated (American Academy of Pediatrics 2019b, 2019c). Therefore, parents in our study may have been more likely to make changes in their home environment because their child(ren) were developmentally approaching new milestones, exposing them to additional injury hazards, during the SAH order.

While the time spent at home because of the COVID-19 SAH order was intended to keep people safe from the spread of the virus it may have motivated the parents in our study to take steps to make their homes safer for their children. We found that during the SAH order, over forty percent (42%) of the sampled parents reported an increase in their home injury prevention practices. Parents who had previously identified unsafe areas in their homes before the SAH were almost three times as likely (2.6) to increase their safety behaviors and about four times more likely to take additional steps to childproof their homes (4.14) and purchase or install safety devices (3.9) during the SAH than those that had not identified unsafe areas in their homes. Previous work has identified a lack of time as a barrier to completing home safety tasks such as installing safety devices (Roberts et al. 2019). The time spent at home during the SAH may have given the parents in our study the opportunity to overcome this barrier and modify unsafe areas that they had previously noticed.

This study had some limitations. First, the participants were recruited via posts made on social media. Those without access to the internet or these social media platforms would not have the ability to participate. Therefore, these results may not be generalizable to all parents with children 6 years old and younger. In addition, the recruitment post was placed on the Nationwide Children’s Hospital’s social media accounts and followers of these accounts may be more interested in pediatric health- and injury-related topics and willing to participate. However, these posts were also shared by individuals via social media and email; thus, the recruitment invitation was able to reach parents that may not have been followers of the Nationwide Children’s Hospital’s social media accounts. Secondly, some survey items were created specifically for this survey and do not have established validity. In addition, participants were asked to self-report their behaviors which can introduce bias in their responses (i.e., what they “believe they are doing” and what they “are actually doing”). However, we do not have any reason to believe that this typical bias was more pronounced in our study compared to other studies collecting self-report data on behaviors. Also, due to the survey platform used, authors were limited in their ability to confirm unique participants. Although the survey platform restricted the ability of multiple survey attempts on one device, we realize that the same user could have completed the survey from a different device that contained a different IP address. Thirdly, the duration of the stay-at-home orders may have varied for our participants. In the USA, the stay-at-home orders were generally from March 1, 2020, until May 31, 2020, but may have varied in duration by location. Additionally, moving homes was associated with the purchasing and installing of safety devices during the SAH order—parents who moved were more likely to complete those safety actions. When moving homes, parents may have needed to assess the new location for safety. The new location may have not included the safety devices that were in their old home thus requiring parents to purchase and install devices to make the new home location safe for their children. Finally, we did not ask the participants to differentiate between a new device installation or a device replacement due to device damage or expiration or because of a move. Future studies could further investigate the differences in safety device purchase and installation for parents of young children before and after they move homes.

Despite these limitations, this study had several strengths, including the ability to recruit a large number of parents with young children quickly and inexpensively via social media and collect information regarding home safety, child injury prevention practices, and safety device acquisition and installation, during a pandemic.

## Conclusions

While the intention of the stay-at-home orders was to slow the spread and keep people safe from the COVID-19 virus, the additional time at home may have helped parents in this study, especially those with young children, increase their injury prevention behaviors and provided parents with the opportunity needed to make their homes safer for their children.

## Supplementary Information

**Additional file 1**: *Safe at Home* Survey: Safe at Home online survey containing 36 questions asking parents of young children (≤ 6 years) about their home safety practices before and during the COVID-19 stay-at-home order.

## Data Availability

The datasets generated and analyzed during the current study are not publicly available but are available from the corresponding author on reasonable request.

## Abbreviations

COVID-19: 2019 Novel coronavirus disease
SAH: Stay-at-home
US: United States
CI: Confidence interval
