# Supplementary material for: Stay-at-home, Safe at Home? A survey of parental home safety practices before and during the COVID-19 pandemic
Source: Inj Epidemiol. 2022 Dec 21;9(Suppl 1):43. doi: 10.1186/s40621-022-00396-4 (PMC9768763; doi:10.1186/s40621-022-00396-4)
Supplement: Supplementary file 1 — Additional file 1: Safe at Home Survey: Safe at Home online survey containing 36 questions asking parents of young children (≤ 6 years) about their home safety practices before and during the COVID-19 stay-at-home order. [file 40621_2022_396_MOESM1_ESM.pdf]

Safe-at-Home - COVID-19 and Injury Prevention

Consent

We are conducting this study because we would like to learn more about you and your home safety practices before and during your state's stay-at-home/shelter-in-place orders as well as your practices today. We are also interested in learning about how parents make decisions about safety preparedness and injury prevention for their children. The purpose of this research is to improve our ability to prevent injuries and understand parent safety behaviors.

If you agree to take part in the study, here is what will happen. Today you will take an online survey. You will be asked to answer questions about yourself and your home safety. This survey will take about 15 minutes. Following the completion of the survey, you will be asked to download the Make Safe Happen, home safety mobile app, and enter a study participant ID that is unique to those that participated in this survey. Information collected from this survey and from the app will remain anonymous.

This study involves little risk to you. It is your choice to be in this study. You may choose to stop being in this study at any time and there will not be any penalty. Also, it will not cost anything to be a part of this study.

By completing the survey, you will have an option to be entered into a drawing and winners will receive a $100 gift card. If you are interested, you will have the option to leave an email address. The email address will not be linked to your survey data.

By continuing on to the survey, I am communicating my consent to participate in this research study being conducted by Dr. Lara McKenzie with the Center for Injury Research and Policy at the Abigail Wexner Research Institute at Nationwide Children's Hospital. I understand the purpose of the study, the procedures that will be followed, and the amount of time it will take. I know that I can choose not to participate without any penalty to me.

I know I can contact Dr. Lara McKenzie at any time by e-mailing her at Lara.McKenzie@nationwidechildrens.org. If I have questions about my rights as a research participant, I can call the Nationwide Children's Hospital Institutional Review Board at (614) 722-2708.

1. Would you like to participate?

- ○ Yes, I would like to participate.
- ○ No thanks.

Safe-at-Home - COVID-19 and Injury Prevention

About You

Please tell us a little bit about you and your family.

2. How old are you?

- Under 18
- 18-24
- 25-34
- 35-44
- 45-54
- 55-64
- 65+

3. Do you currently have a child 6 years old or younger that lives with you in your home, most of the time?

- Yes
- No

4. What are the ages of the children (6 years or younger) that currently live in your home?

	1	2	3	4 or more	Not Applicable
0-11 months					
1 year					
2 years					
3 years					
4 years					
5 years					
6 years					
7 years or older					

5. In what state or U.S. territory do you live?

6. In what city do you live?

7. In what ZIP code is your home located? (enter 5-digit ZIP code; for example, 00544 or 94305)

8. Since the COVID-19 pandemic emerged both locally and globally, many countries have imposed lockdown policies, such as stay-at-home orders, work-at-home orders for non-essential employees, closures of schools and non-essential businesses, social distancing, wearing face masks, etc. Which of the following policies have been imposed where you live (check all that apply)?

- ☐ Stay-at-home order
- ☐ Social distancing
- ☐ Restrictions on large gatherings
- ☐ Wearing facemasks in public
- ☐ Closure of non-essential business
- ☐ I don't know
- ☐ Other (please specify)

9. Which of the following policies are you currently practicing (check all that apply)?

- ☐ Staying at home
- ☐ Social distancing
- ☐ Limiting contact with friends/family
- ☐ Avoiding large gatherings
- ☐ Wearing facemasks in public
- ☐ Frequent hand washing
- ☐ Other (please specify)

10. Is the area where you currently live under a stay-at-home or shelter-in-place order (to prevent the spread of COVID-19)?

- ○ Yes
- ○ Not at this time
- ○ I don't know

11. Have you ever downloaded the Make Safe Happen home safety mobile app?

- ○ Yes, I use this app
- ○ Yes, I downloaded the app but do not use it regularly
- ○ Yes, I downloaded the app but never used it
- ○ No

Safe-at-Home - COVID-19 and Injury Prevention

BEFORE Stay-at-Home or Shelter-in-Place (January/February 2020)

Please think about your life and your home BEFORE the stay-at-home or shelter-in-place order to prevent the spread of COVID-19. While answering these questions, think of your home in January or February of 2020.

12. BEFORE the stay-at-home order, how safe (in terms of preventing child injuries) did you consider your home to be for your child(ren)?

Not safe, a lot of room for improvement	Safe but room for improvement	Extremely safe

13. BEFORE the stay-at-home order, our home had the following safety products...

	Yes	No	Not applicable
Smoke Alarms	○	○	○
Carbon Monoxide Alarms	○	○	○
Cabinet locks or latches	○	○	○
Stair gates, room gates or barriers	○	○	○
TV wall mount or anti-tip straps	○	○	○
Door knob covers	○	○	○
Window stops or locks	○	○	○
Other safety products	○	○	○

14. BEFORE the stay-at-home order, had you noticed areas in your home that may be unsafe for your child?

- ○ Yes
- ○ No
- ○ I don't know

Safe-at-Home - COVID-19 and Injury Prevention

DURING the Stay-at-Home or Shelter-in-Place (March/April/May)

Please think about your life and your home DURING the stay-at-home or shelter-in-place order to

prevent the spread of COVID-19. While answering these questions, think of your home in MARCH/APRIL/MAY of 2020, even if there was no official stay-at-home order in place in your area.

15. DURING the stay-at-home order, how safe did you consider your home to be for your child(ren)?

Not safe, a lot of room for improvement	Safe but room for improvement	Extremely safe

16. DURING the stay-at-home order, the amount of time I spent in the home…

- Increased
- Decreased
- Did not change

17. DURING the stay-at-home order, the amount of time my CHILD(REN) spent in the home…

- Increased
- Decreased
- Did not change

Safe-at-Home - COVID-19 and Injury Prevention

Since the Beginning of Stay-at-Home

Please think about your life and your home since the beginning of the stay-at-home or shelter-in-place order to prevent the spread of COVID-19. While answering these questions, think of your home in since MARCH through today.

18. Since the beginning of the stay-at-home order or start of COVID-19 in the US, have you taken additional steps to child proof your home?

- Yes
- No
- Prefer not to answer

19. Since the beginning of the stay-at-home order or start of COVID-19 in the US, have you noticed additional areas in your home that may be unsafe for your child?

- Yes
- No
- I don't know

20. Since the beginning of the stay-at-home order or start of COVID-19 in the US, I have had the following safety products installed in my home...

	Yes	No	Not applicable
Smoke Alarms	◯	◯	◯
Carbon Monoxide Alarms	◯	◯	◯
Cabinet locks or latches	◯	◯	◯
Stair gates, room gates or barriers	◯	◯	◯
TV wall mount or anti-tip straps	◯	◯	◯
Door knob covers	◯	◯	◯
Window stops or locks	◯	◯	◯
Other safety products	◯	◯	◯

21. Since the beginning of the stay-at-home order or start of COVID-19 in the US, have you purchased or installed any safety devices for your home?

◯ Yes

◯ No

◯ I don't know

22. Compared to prior to the stay-at-home order, have your safety actions/behaviors (e.g. lock up medicines, turn pot handles to the back of the stove when cooking) changed during the stay-at-home order?

◯ Yes, my safety actions have INCREASED

◯ Yes, my safety actions have DECREASED

◯ No, my safety actions have not changed

23. Since the beginning of the stay-at-home order, have you moved?

◯ Yes

◯ No

Safe-at-Home - COVID-19 and Injury Prevention

Injuries at Home

24. Compared to before the stay-at-home order, DURING the stay-at-home order, the number of injuries to my child(ren) that occur at home has…

- Increased
- Decreased
- Did not change
- We have not had an injury occur in our home

25. Think about life BEFORE the stay-at-home order or COVID-19. Please tell us how you felt about caring for your children...

	Strongly agree	Agree	Somewhat agree	Disagree	Strongly disagree
I need to pay close attention to my child(ren).	○	○	○	○	○
I feel I can step away briefly.	○	○	○	○	○
I feel my child(ren) can self play and require little attention from me.	○	○	○	○	○
I get distracted while caring for my child(ren).	○	○	○	○	○
I feel tired.	○	○	○	○	○
I feel overwhelmed.	○	○	○	○	○
I feel it is hard to give me child(ren) my full attention.	○	○	○	○	○

26. Think about life DURING the stay-at-home or COVID-19. Please tell us how you felt about caring for your children...

	Strongly agree	Agree	Somewhat agree	Disagree	Strongly disagree
I need to pay close attention to my child(ren).	◯	◯	◯	◯	◯
I feel I can step away briefly.	◯	◯	◯	◯	◯
I feel my child(ren) can self play and require little attention from me.	◯	◯	◯	◯	◯
I get distracted while caring for my child(ren).	◯	◯	◯	◯	◯
I feel tired.	◯	◯	◯	◯	◯
I feel overwhelmed.	◯	◯	◯	◯	◯
I feel it is hard to give me child(ren) my full attention.	◯	◯	◯	◯	◯

27. While caring for my children it is easy to get distracted.

Disagree Neutral Agree

28. For each statement shown below, how confident are you?

	Extremely confident	Very confident	Moderately confident	Slightly confident	Not at all confident
I am ______ that I do everything that is recommended by home safety experts.	○	○	○	○	○
I am ______ that I can take actions to make my home as safe as possible.	○	○	○	○	○
I am ______ that I know the most common home injury hazards.	○	○	○	○	○
I am ______ that I can purchase the exact home safety products and devices I need.	○	○	○	○	○
I am ______ that I can correctly install the safety products and devices in my home.	○	○	○	○	○
I am ______ that I know what to do to make my home as safe as possible.	○	○	○	○	○
I am ______ that taking safety actions will keep my child from being injured in and around my home.	○	○	○	○	○

29. While in your home, have any of your children been injured badly enough to be seen by a doctor or nurse in an office, clinic or emergency department? Check all that apply.

- ☐ Yes, before the stay at home order
- ☐ Yes, during the stay at home order
- ☐ Yes, after the stay at home order
- ☐ No
- ☐ Prefer not to answer

Safe-at-Home - COVID-19 and Injury Prevention

Demographics

Please tell us a little more about yourself.

30. Please tell us about your child care status (for your child(ren) 6 years old or younger). During the day, my child(ren) are at...(Check all that apply)

	Before the stay-at-home order	During the stay-at-home order	Today	Prefer not to answer
School (in person)	☐	☐	☐	☐
A child care facility	☐	☐	☐	☐
An in home child care	☐	☐	☐	☐
In my home with me, a family member, or a nanny	☐	☐	☐	☐
A family member or friend's home	☐	☐	☐	☐

Other (please specify)

31. Please us the status of your employment. (Check all that apply)

	Before stay-at-home order	During the stay-at-home order	Today	Prefer Not to Answer
Employed full time	☐	☐	☐	☐
Employed part time	☐	☐	☐	☐
Unemployed	☐	☐	☐	☐
Stay at home parent	☐	☐	☐	☐
Student	☐	☐	☐	☐
Retired	☐	☐	☐	☐
Unable to work	☐	☐	☐	☐

Other (please specify)

32. Were you able to work from home during the stay-at-home order?

- ○ Yes
- ○ Somewhat
- ○ No
- ○ Other (please specify)

33. Are you or any other adult living in your home considered an essential worker (i.e. health care provider, first responder, grocery workers, etc)?

- Yes
- No
- I don't know

34. Did you homeschool any school age children during the stay at home order?

- Yes
- No
- I homeschooled prior to the stay-at-home order

35. Thinking of your household's total income, how would you say that your household is able to make ends meet?

	Before Stay at Home Order	During the Stay at Home Order	Today
With great difficulty	☐	☐	☐
With difficulty	☐	☐	☐
We get by	☐	☐	☐
Easily	☐	☐	☐
Very easily	☐	☐	☐
Prefer not to answer	☐	☐	☐

36. How much of a burden has the coronavirus outbreak been on your household’s finances?

- A substantial burden
- A moderate burden
- Not much of a burden
- Not a burden at all
- Prefer not to answer
